# Commentary on: “Tissue engineering: How to build a heart”

**DOI:** 10.3389/fphys.2015.00084

**Published:** 2015-03-19

**Authors:** Valentina Di Felice

**Affiliations:** ^1^Department of Experimental Biomedicine and Clinical Neurosciences, University of PalermoPalermo, Italy; ^2^Dipartimento di Medicina e Terapie d'Avanguardia, Strategie Biomolecolari e Neuroscienze, Istituto Euro-Mediterraneo di Scienza e TecnologiaPalermo, Italy

**Keywords:** cardiac tissue engineering, decellularization, stem cells, cardiac progenitor cells

Decellularization and recellularization of hearts from newly dead donors is the latest fashion in cardiac tissue engineering. The first paper came out in 2008 in Nature Medicine (Ott et al., 2008), and news has been recently published in Nature again in July 2013 (Maher, 2013).

Brendan Maher in this paper summarizes and comments on the latest important results on decellularization of a human heart and explains the steps that are necessary to build a heart from a decellularized organ. Two sources may be used to obtain a decellularized heart: human and pig heart. Another issue to resolve is the time of decellularization, since the detergents used may also destroy the architecture of the organ and adhesion molecules useful for the colonization of the newly introduced cells. The author also highlights two main problems, which cell type to introduce to the decellularized organ and how to establish and maintain the organ's ability to beat. Many researchers use a mixture of stem and progenitor cells from the blood vessels and from the heart; Ott and colleagues use induced Pluripotent Stem (iPS) cells. After having chosen the best progenitor cell, another problem is to let the introduced cells distribute uniformly in the decellularized scaffold, and to let them grow as they are in a natural environment. A way to improve cell growth in these scaffolds is to use bioreactors that electrically stimulate the heart and mimic forces of a beating heart. The most difficult step in implanting decellularized/recellularized organs is to connect them to the host body. The first problem is to connect vascularization of the new organ with the one of the host living animal. Ott's team and others have implanted reconstructed hearts into rats in parallel with other organs, but even if researchers have fed the organs with blood and get them to beat for a while, none of the hearts has been able to continue for long. At the moment researchers are able to implant recellularized hearts only in small animals.

In the last 6 years many research groups tried to remove cells from a dead organ and to repopulate it with stem cells or multipotent cells, immunologically matched to the patients, or decellularize the entire organ preserving chemotactic and pro-angiogenic properties, so that they can be successfully used for clinical tissue engineered airway clinical replacements in infants (Baiguera et al., 2010).

It looks simple for tubular structures as the trachea, but extremely difficult for more complex organs as the heart. This is a fine pump, working 24 h a day, with valves, tendinous chords, chambers, heart walls and septa made up of different types of specialized cells, working cardiomyocytes or cardiomyocytes of the conductive system. The cells needed to re-populate the entire heart probably derive from the same cardiac progenitor cell (Di Felice and Zummo, 2013), but until today it is very difficult to obtain both a good number of these cells and to differentiate them into one or the other of the four types of cells which populate the heart.

Difficulties in re-building a heart are: recreating the vasculature of the heart, since the cardiac tissue is supplied by intricate networks of capillaries difficult to reproduce; eliminating residues of the detergents used, which may influence stem cell growing and differentiation; finding suitable donor hearts, because available organs are often damaged by diseases or infectious agents.

Other animal sources would be beneficial, Ott's team is trying to use decellularized porcine organs to substitute damaged human ones. Anyway a discussion on the use of porcine substitutes is still open for debate. The pig is a good candidate because it is anatomically and physiologically similar to man, but a violent immune reaction involving the complement system occurs, leading to hyperacute rejection (HAR). Many attempts are still in progress to produce transgenic pigs for one of the regulators of complement activation (RCA), or other molecules of the complement system (Lavitrano and Frati, 1999).

On the other hand, one of the main issues encountered in cardiac tissue engineering arises from the difficulty to realize scaffolds able to match the elasto-mechanical properties of the heart wall in which the artificial construct is thought to be integrated. In this respect the “elastic” response of the scaffold should be tailored and assessed in advance, with the aim to both meet the physiological mechanical properties of the heart wall and the eventual structural needs emerging after a myocardial damage.

A successful approach to cardiac tissue engineering should aim at developing scaffolds that mimic the elasto-mechanical properties of the heart wall, able to promptly respond to the hemodynamic forces of the blood and resembling the dynamic features of the heart wall. Moreover, recently it has been demonstrated how hemodynamic forces regulate development of the conductive system (Bressan et al., 2014), and it has been suggested that the biomechanics forces present in the heart may regulate cardiac development (Lindsey et al., 2014). With this in mind, the elastic anisotropism, known to characterize the mechanical properties of the heart, may be measured in an explanted heart, and the obtained parameters may be taken into account in order to produce a tailored biomaterial that would exhibit a full compatibility not only at the biological level but also for the structural and mechanical asset of the organ. A decellularized heart may represent the natural scaffold which may resemble the fine elasto-mechanical properties of such a complex organ.

Considering the difficulties in finding human donor hearts, and the need to recreate the elasto-mechanical properties of the heart wall, the best solution would be to design and print tolerated scaffolds on the shape of the heart of a patient. Customized scaffolds three-dimensionally printed on the radiological images obtained from the patient.

An alternative to decellularized organs is the use of *de-novo* cellular-derived matrices (CDM) to create customized scaffolds and organs (Fitzpatrick and Mcdevitt, 2015). Structures obtained from the combination of natural matrices may overcome many problems encountered in decellularized organs, such as the presence of detergents and the low availability of donor hearts. Moreover, CDM may be used in three-dimensions printers to obtain a personalized scaffold.

## Conflict of interest statement

The author declares that the research was conducted in the absence of any commercial or financial relationships that could be construed as a potential conflict of interest.
